# Netrin-1’s unique surface pocket for a potential anti-cancer drug-binding target

**DOI:** 10.1093/procel/pwaf099

**Published:** 2025-11-14

**Authors:** Jia-huai Wang

**Affiliations:** Dana-Farber Cancer Institute, Harvard Medical School, Boston, MA 02215, United States

Netrin-1 was the first axon guidance cue identified (Serafini et al., 1994). It is an embryonic, secreted, laminin-related glycoprotein that plays diverse functions in cell biology, including neural development, organogenesis of other systems, and cell survival (Boyer and Gupton, 2018; Lai Wing Sun et al., 2011). Expressed mainly during embryonic development, netrin-1 has been shown to be re-expressed by cancer cells in a large proportion of human neoplasms, which stimulates tumor growth (Cassier et al., 2023). Netrin-1’s primary receptor, deleted in colorectal cancer (DCC), was initially discovered as a putative tumor suppressor gene in colon cancer, as its name implies (Fearon et al., 1990), and was later established as a netrin-1 receptor (Chan et al., 1996; Keino-Masu et al., 1996). Interestingly, DCC belongs to a class of receptors termed “dependence receptors.” In the presence of cognate ligands, such as netrin-1, they transduce signals needed for cell survival, to guide axons for instance. In the absence of ligands like netrin-1, these receptors do not stay inactive. Rather, they elicit an apoptotic signal that kills the cells (Goldschneider and Mehlen, 2010). There is now evidence that netrin-1 and its receptor DCC define a new mechanism of tumorigenesis. The binding of netrin-1 to its receptor inhibits tumor apoptosis (Arakawa, 2004). Interfering with netrin-1/receptor interaction might prove to be a good strategy for cancer treatment. Indeed, recent data demonstrate that pharmacological targeting of netrin-1 using NP137, a netrin-1-blocking monoclonal antibody, manifests a safe and effective strategy in primary mouse and human tumors, as shown by current phase II clinical trials for the treatment of different solid tumors (Lengrand et al., 2023).

Figure 1A is a schematic of the domain organization of netrin-1, DCC, and another guidance cue molecule, draxin, that modulates the netrin-1/DCC interaction (Islam et al., 2009; Liu et al., 2018). The figure also briefly shows the interactions among these three partners as reviewed in (Meijers et al., 2020). Netrin-1 consists of a large laminin-like domain (LN), three epidermal growth factor (EGF) domains, and a C-terminal NTR domain. DCC is composed of four immunoglobulin (Ig)-like domains, followed by six fibronectin type III domains (FN) as its extra-cellular portion. DCC’s domains FN4, FN5, and FN6 are involved in netrin-1-binding. Its FN4 binds to netrin-1’s LN domain. One FN5-FN6 goes to netrin-1’s EGF1-EGF2 (not shown in Fig. 1A), and another FN5 contacts the EGF3 of the same netrin-1 molecule (Finci et al., 2014; Xu et al., 2014). Draxin comprises a large unstructured N-terminal polypeptide of 260 residues that contains the 22-residue conserved hydrophobic sequence (P22) that binds to netrin-1’s EGF3 and an approximately 90-residue cysteine knot domain at the C terminus (Draxin-C) that binds to DCC’s Ig4 domain (Liu et al., 2018) (Fig. 1A). The complicated modulation of interactions among netrin-1, DCC, and draxin synergizes signaling and adhesion for axon guidance (Meijers et al., 2020).

**Figure 1.**
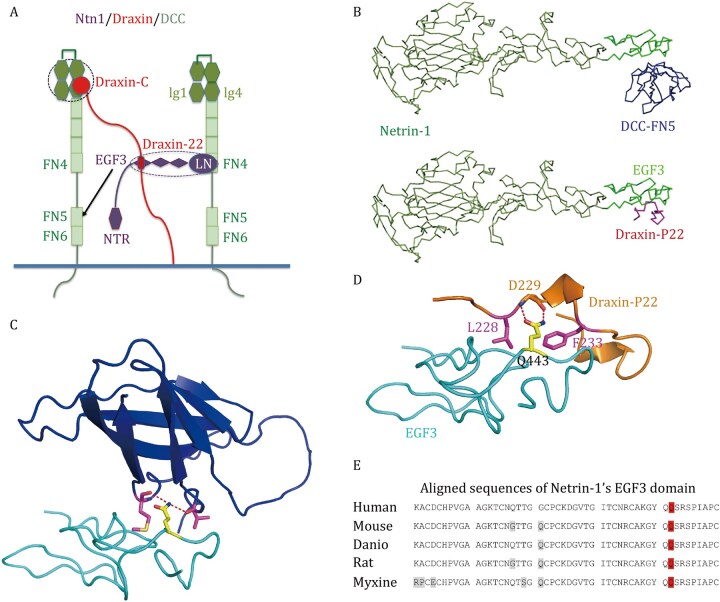
The netrin-1 interaction. (A) Schematic of domain organization of netrin-1 (dark blue), DCC (the dark green for four Ig-like domains and light green for six FN domains), and draxin (red); (B) The sideview of netrin-1 interacting with DCC-FN5 (PDB 4URT) in the upper panel and with draxin-P22 (PDB 6FKQ) in the lower panel; (C) Q443-centered interaction between netrin-1’s EGF3 domain and DCC-FN5 domain; (D) Q443-centered interaction between netrin-1’s EGF3 domain and a draxin-P22 motif; (E) Aligned sequences of Netrin-1 EGF3 domain from 5 species. The red-shaded Q443 is invariant. The grey-shaded residues are different from that of human Netrin-1.

One notable feature of Fig. 1A is that DCC and draxin share their binding site at the same netrin-1’s EGF3 domain, which contributes to the competitive modulation as discussed in the review (Meijers et al., 2020). However, for this short article, we are looking beyond netrin-1’s fascinating role in neuroscience to its significant potential in anti-cancer drug design. Figure 1B illustrates the structures of the binding of DCC-FN5 and draxin-P22 to EGF3 (PDB 4URT and PDB 6FKQ, respectively) in the same sideview. What’s intriguing is how the two entirely different structural entities, FN5 and P22, are able to bind to the same small EGF domain on netrin-1. What unique structural feature on the EGF3 domain surface defines these bindings? Most importantly, can researchers exploit this feature as a potential anti-cancer strategy and design small molecules that block netrin-1 from interacting with its receptors? When we carefully scrutinize the center of EGF3/DCC-FN5 and EGF3/draxin-P22, as in Fig. 1C and 1D, respectively, a common structural feature manifests. In both structures, the EGF3 residue Q443 plays a critical role in pulling the binding partners into place. In Fig. 1C, the amide nitrogen atom of Q443 sidechain forms bifurcated hydrogen bonds to mainchain carbonyl oxygen atoms of M933 and V848 (there are other hydrogen bonds from the amide oxygen of Q443 to EGF3 domain, but for clarity, they are not shown in the figure), which brings the hydrophobic sidechains of FN5’s M933 and V848 into contact on the EGF3 surface for binding. Similarly, in Fig. 1D, the amide group of Q443 forms bidentate hydrogen bonds to the mainchain carbonyl and amide groups of draxin-P22’s D229, which pull P22’s neighboring hydrophobic residues L228 and F233 into the same area on the EGF3 surface for binding. Figure 2A and 2B give a top view of the two EGF3-bindings by FN5 and P22, respectively. The EGF3 domain is displayed as a surface representation with blue and red color for positive and negative charges, respectively, while the white color represents a neutral and hydrophobic surface. One remarkable common characteristic of the two structures is that the two binding sidechains—M933 and V848 in DCC-FN5 and L228 and F233 in draxin-P22—extend into a gourd-shaped hydrophobic pocket in a very similar fashion, with the EGF3’s Q443 sitting at the waist of the pocket. What we now term the “Q443-pocket” is a well-described “hotspot” for protein-protein interaction.

**Figure 2.**
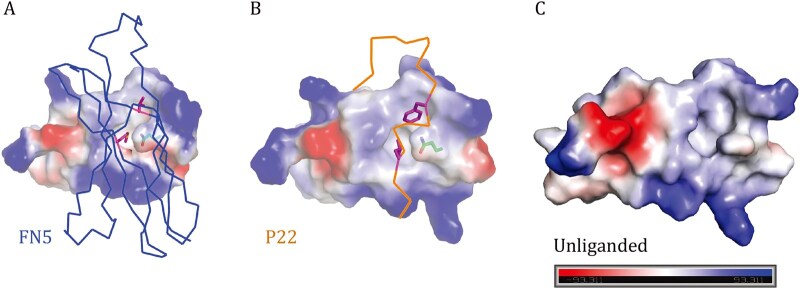
The top view of the Q443-pocket of the EGF3 domain in surface representation (blue and red for positive and negative charges, respectively, and white for a neutral and hydrophobic surface). (A) netrin-1’s FN5 binding. See how FN5’s hydrophobic sidechains of V848 and M933 extend into a gourd-shaped pocket on the EGF3 surface with EGF3’s Q443 sitting at the waist of the pocket; (B) Draxin’s P22 binding. P22’s hydrophobic sidechains of F233 and L228 extend into the same gourd-shaped pocket on the EGF3 surface with Q443 on the edge; (C) The unliganded EGF3 domain from Gallus gallus netrin-1 (PDB 9D77). The characteristic Q443-pocket is clearly seen. The electrostatic potential scale is shown underneath.

“Hotspots” were first defined in the structural and functional studies of the interactions between a growth hormone and its receptor, which indicated that only a small fraction of contact residues contribute to major binding energy as a “functional epitope” (Clackson and Wells, 1995). The hotspot residues tend to be concentrated in small hydrophobic pockets, comparable to the size of a small molecule that could dynamically adjust to bind a drug-like molecule (Arkin et al., 2014). The so-called “druggability” of a protein interaction site is a property encoded on a protein surface through its propensity to form pockets (Johnson and Karanicolas, 2013). Very often, the complemented pockets are pre-organized in the unbound state (Li et al., 2004). The Q443 pocket we identified on netrin-1’s EGF3 domain surface bears the following traits that make it a particularly good candidate for potential medicinal drug design. First, this Q443-pocket facilitates the binding of two partners with entirely different structures, which implies that there is a common focused structural pattern for the pocket recognition. Second, Fig. 2C gives a surface presentation for a newly released unliganded structure from a Gallus gallus netrin-1 EGF3 domain (PDB 9D77). Apparently the Q443 pocket is indeed pre-organized in the apo-state, which suggests it may be possible to carry out a small-molecule search for a binder. Third, a very unique feature of the Q443 pocket is not just its hydrophobic nature within the pocket for accommodating two hydrophobic sidechain groups, but also that it has an amide sidechain group of Q443 at the edge of the pocket to render specific hydrophilic interaction to a potential binder. It is particularly interesting to note that the residue Q443 is very conserved for netrin-1 from human to myxine (Fig. 1E).

Traditionally, the concept of high-throughput screening of a large, diverse small-molecule library that first appeared in the mid-1980s now forms one of the cornerstones of modern drug discovery (Wildey et al., 2017). It should aid the search for drug-like binders of the targeted Q443-pocket that we discussed above. On the other hand, with limited chemical space available in search libraries, generative AI offers a fresh approach to *de novo* drug design. As pattern matching for chemical design becomes more routine (Schneider et al., 2020), we might be able to find small molecules to fit into the Q443 pocket with its unique pattern. The hope is that structural investigation of netrin-1 biology and the Q443 pocket may one day lead to potential anti-cancer drug discovery.

## Data Availability

The original structure work has been published in *Neuron*, 2018; 97: 1261-1267.
